# Delphi study to derive expert consensus on a set of criteria to evaluate discharge readiness for adult ICU patients to be discharged to a general ward—European perspective

**DOI:** 10.1186/s12913-022-08160-6

**Published:** 2022-06-13

**Authors:** Maike Hiller, Maria Wittmann, Hendrik Bracht, Jan Bakker

**Affiliations:** 1 Department of Intensive Care Adults, Erasmus MC University Medical Center, Rotterdam, The Netherlands; 2 Department of Hospital Patient Monitoring, Clinical Services, Philips Medizin Systeme Böblingen GmbH, Böblingen, Germany; 3 Department of Anesthesiology and Intensive Care Medicine, University Hospital Bonn, Bonn, Germany; 4 Central Emergency Medicine Services and Department of Anesthesiology and Intensive Care Medicine, University Hospital Ulm, Ulm, Germany; 5 New York University School of Medicine and Columbia University College of Physicians & Surgeons, New York, USA; 6 Department of Intensive Care, Pontificia Universidad Catolica de Chile, Santiago, Chile

**Keywords:** Intensive care unit, Adult patient, Discharge, Assessment, Care transition, Objective criteria, Checklist, General ward, Multidisciplinary team

## Abstract

**Background/purpose:**

Discharge decisions in Intensive Care Unit (ICU) patients are frequently taken under pressure to free up ICU beds. In the absence of established guidelines, the evaluation of discharge readiness commonly underlies subjective judgements. The challenge is to come to the right decision at the right time for the right patient. A premature care transition puts patients at risk of readmission to the ICU. Delayed discharge is a waste of resources and may result in over-treatment and suboptimal patient flow. More objective decision support is required to assess the individual patient’s discharge readiness but also the current care capabilities of the receiving unit.

**Methods:**

In a modified online Delphi process, an international panel of 27 intensive care experts reached consensus on a set of 28 intensive care discharge criteria. An initial evidence-based proposal was developed further through the panelists’ edits, adding, comments and voting over a course of 5 rounds. Consensus was defined as achieved when ≥ 90% of the experts voted for a given option on the Likert scale or in a multiple-choice survey. Round 1 to 3 focused on inclusion and exclusion of the criteria based on the consensus threshold, where round 3 was a reiteration to establish stability. Round 4 and 5 focused on the exact phrasing, values, decision makers and evaluation time frames per criterion.

**Results:**

Consensus was reached on a standard set of 28 ICU discharge criteria for adult ICU patients, that reflect the patient’s organ systems ((respiratory (7), cardiovascular (9), central nervous (1), and urogenital system (2)), pain (1), fluid loss and drainages (1), medication and nutrition (1), patient diagnosis, prognosis and preferences (2) and institution-specific criteria (4). All criteria have been specified in a binary decision metric (fit for ICU discharge vs. needs further intensive therapy/monitoring), with consented value calculation methods where applicable and a criterion importance rank with “mandatory to be met” flags and applicable exceptions.

**Conclusion:**

For a timely identification of stable intensive care patients and safe and efficient care transitions, a standardized discharge readiness evaluation should be based on patient factors as well as organizational boundary conditions and involve multiple stakeholders.

**Supplementary Information:**

The online version contains supplementary material available at 10.1186/s12913-022-08160-6.

## Background

A growing group of elderly, more fragile and multimorbid patients will increase the future need for intensive care capacities. To meet this demand, adding more intensive care unit (ICU) beds is often not an option due to financial limitations and the lack of specialized and highly skilled care givers to staff the beds. Therefore, optimizing the utilization of given ICU resources is a high priority for hospital management to avoid bottleneck situations. Capacity strain is often created by pending discharges and flow delays which could account to 15–25% of the total ICU length of stay (LOS) [1, 2]. On the other hand, high census levels can cause premature discharges resulting in unstable patients at lower levels of care together with an overestimation of the receiving ward capacities. That ultimately results in readmissions and even increases capacity strain, LOS, mortality and costs [3–5]. Optimal patient outflow can be achieved by identifying those patients early that are stable enough to transition to the next lower level of care and keeping boarding time to a minimum through close cooperation with the receiving unit [1, 5, 6]. Discharging the right patient at the right time reduces LOS, readmission rates, and costs, where an inappropriate discharge will achieve the opposite and increase risk of mortality [7–9]. Until today, discharge readiness assessment is often rushed, subjective and untransparent, and based on a limited amount of available aggregated data and decision criteria. The need for a comprehensive proposal of discharge criteria for adult ICUs that is widely applicable in daily clinical practice throughout Europe has been phrased in a variety of studies [5, 9–13], and is even more relevant today in light of the ongoing COVID-pandemic and extremely strained ICU capacities. But what does it take to identify stable patients timely, minimize their boarding time and ensure safe and efficient care transitions? Basically, the set of criteria for an objective evaluation of patient discharge readiness should satisfy two purposes: First, patient specific criteria such as patient status, interventions and medications, diagnosis and prognosis should indicate a stable state of the patient for at least the next 48 h that allows safe discharge with a minimized risk of readmission. Second, the set of criteria should incorporate system-specific criteria such as nursing workload related criteria at the discharging and the receiving unit, and institutional factors such as available technical infrastructure, skill sets, patient/nurse ratios, protocols and processes.

The absence of such a holistic discharge readiness evaluation tool was the motivation behind this study. The study group condensed evidence-based criteria and recommendations, structured and referenced them in a table format as a first proposal for a set of discharge criteria. This proposal was then subject to a 5-level Delphi study involving a multi-professional panel of European intensive care experts to reach consensus on a standard set of discharge criteria. Providing a consented and standardizable set of criteria for use in daily clinical practice should provide a holistic view to the interdisciplinary care team on individual patient discharge readiness and organizational capabilities. Further, it should guarantee equity in care provision by improving objectivity and comparability in clinical decision making, and increase quality of care transitions and efficient use of ICU capacities in the interest of the patient and the society [14].

## Methods

With the study aim to reach consensus on a standardized set of ICU discharge criteria, the research group selected a modified online Delphi process. The Delphi method was chosen as it is a suitable research tool, specifically in areas where there is limited scientific evidence, lack of agreement, incomplete knowledge or uncertainty and conclusions are heavily relying on expert opinion [15–18]. In this case, it should help to build on the limited scientific evidence for established and well-defined ICU discharge criteria that was identified by the previously performed scoping literature review [19]. Further, the Delphi technique had four main characteristics that suited the objective of this study: anonymity between participants through a non-face-to-face format, iteration with controlled feedback of group opinion, statistical aggregation of group responses and expert input [18]. The study was realized via an online voting platform designed for Delphi studies (welphi.com) to include a geographically spread panel of experts and to allow participation in an asynchronous, online, participatory and interactive way, at comparatively low cost and time investment.

Generally, the Delphi study was conducted in three stages: 1.) Scoping literature review, criteria preselection and panelist recruitment, 2.) Online Delphi process (detailed process description in online supplement b, doc. 2), 3.) Conclusion on final results.

In the 1^st^ stage, the investigators derived a preselection of criteria from the earlier performed scoping literature review [19] that was then reviewed by selected experts for suitability and comprehensiveness. Items, values and ranges were added or edited where applicable. Scientific evidence was referenced per criterion and a first proposal of an ICU discharge criteria checklist was structured by criteria categories (online supplement a, doc. 1, tab. S1). Potential experts were identified based on a combination of proven research activities (topic-related publications in Pubmed and on ResearchGate, topic related congress presentations (ESICM, ISICEM) and through peer recommendation of being a practice specialist in the specific field. In addition, the investigators aimed for an expert panel representing a diversity of European countries and healthcare systems as well as a balance in professions. In this context, the selected and approached experts mainly work in Western European and North American countries with high standards of care, although in different healthcare system settings. Email invitations with background and introduction to the study were sent out to the potential panelists. Upon acceptance to participate, 28 enrolled participants (17 ICU doctors, thereof 2 female, and 11 ICU nurses, thereof 6 female), representing 12 different healthcare systems, received a pre-read document (online supplements a, doc. 1) and their personalized sign-in credentials to access the online Delphi tool.

**Fig. 1:**
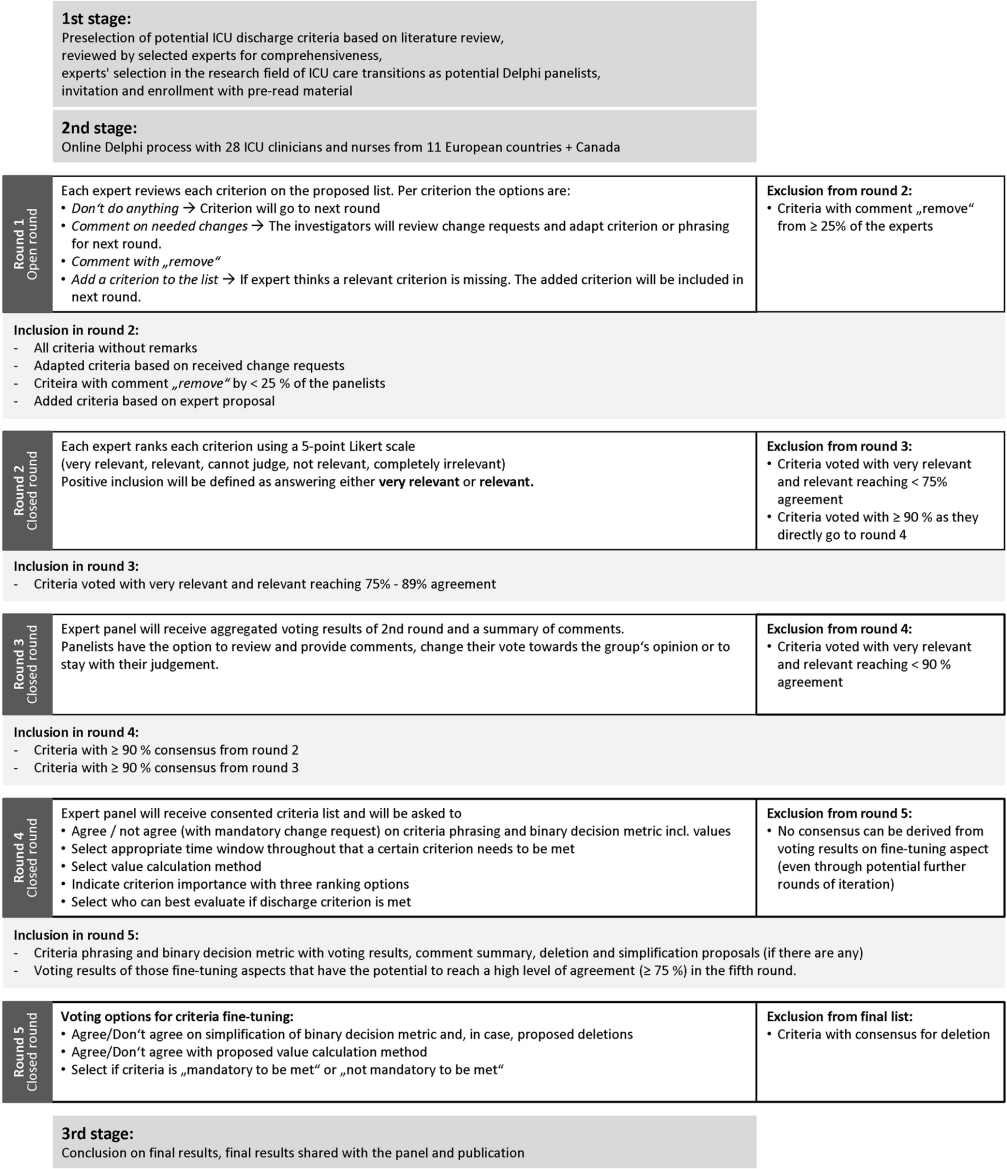
Graphical illustration of the Delphi process

In the online Delphi process (2^nd^ stage), expert group consensus was built through an iterative process that used systematic progression through five rounds of voting on questions, statements, or criteria in this case [20]. The individual rounds of the process were set-up based on available literature on Delphi studies in similar settings [15, 17, 18, 21, 22], process reviews with appointed methodologists and the technical support team of the online Delphi platform. The enrolled panelists voted on every criterion for inclusion in a standard set of discharge criteria that is necessary and suitable to evaluate individual patient’s discharge readiness for adult patients in any type of European intensive care setting and not specific to any individual disease process or specialty. The investigators did not actively participate in the online Delphi process, but reviewed, summarized and discussed the results after each round in order to set up the following rounds. Consensus was defined through agreement on proposed criteria by ≥ 90% of the panelists [22, 23]. For the first three rounds, exclusion of criteria from subsequent rounds was defined by reaching < 75% of agreement per criterion with the cut-off value oriented on comparable research [23]. In round 1, agreement for inclusion for round 2 was defined per criterion through not editing / leaving as is, editing, or adding a criterion to the list. The remark “removal*”* per criterion was counted as disagreement and a criterion would have been removed from the list if ≥ 25% of the experts voted for removal in round 1. In round 2, agreement for further inclusion of a criterion in the list was defined as answering either *very relevant* or *relevant* per criterion on the provided Likert scale (5 values: “very relevant”, “relevant”, “cannot judge”, “not relevant”, “completely irrelevant”). Criteria that met consensus of ≥ 90% through answers of “very relevant” or “relevant” were already approved to enter round 4. Criteria that reached agreement between 75 – 89% on being “very relevant” or “relevant”, went into round 3, where experts were confronted with the voting results on group level compared to their own results and outliers had the chance to change their vote towards the groups opinion. In round 4, only those criteria having reached consensus in round 2 and the additional criteria finally having reached consensus of ≥ 90% in round 3, went through further finetuning on criteria importance rank, criteria evaluation time frames, specific values, and calculation method as well who from the care team could best evaluate per criterion if it has been met. For round 5, the panelists received a statistical summary report of the group voting on criteria phrasing, importance ranking, evaluation time windows, criteria calculation, and preferred decision makers per criterion. Further, they were asked to agree / disagree on the final criteria phrasing, value calculation method and criteria importance ranking. The aspects “criteria evaluation time frame” and “who could best evaluate if a specific criterion has been met” where excluded from further voting due to the heterogeneity of the round 4 results (online supplements c, results round 4). Based on the voting results and provided comments, the investigators derived the final list of discharge criteria, shared the results with the expert panel and prepared the study manuscript for publication. An overview of the set-up of the five rounds is given in Fig. 1.

In the 3rd stage, based on the voting results of the 5th round and the provided comments, the team of investigators concluded on the final list of 28 ICU discharge criteria with their binary decision metric (values for Fit for ICU discharge and Needs further intensive care therapy / monitoring). Where appropriate—the preferred value calculation method to evaluate discharge was proposed, and a criterion importance ranking was provided with exceptions when the mandatory to be met—requirement could be overruled.

## Results

**Fig. 2:**
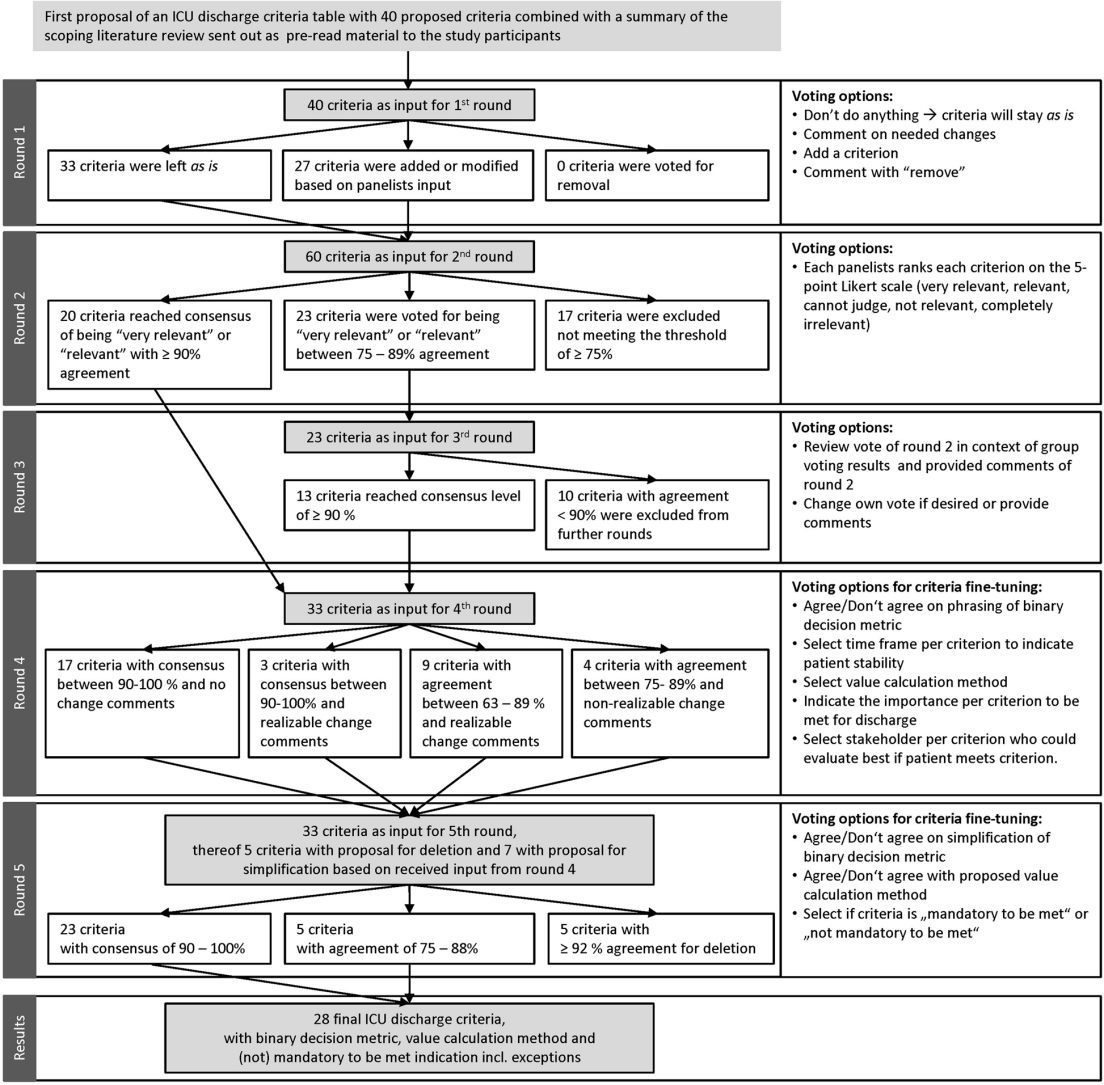
Results of the different rounds in the online Delphi process

Of the 54 experts (33 medical doctors and 21 nurses) approached, 17 doctors (2 female) and 11 nurses (6 female) agreed to participate. One participant left the panel after the second round. The final panel represented 12 different healthcare systems (11 European, one Canadian) (online supplements c, fig. S1) and brought in experience from different ICU types and sizes and professional backgrounds: More than half of the participants worked in mixed ICUs (mixed ICUs (18), medical ICUs (2), surgical ICUs (3), other (1), no info (4)). Different ICU sizes were equally represented (4–12 bed ICUs (8), > 12 – 24 bed ICUs (7), > 24 bed ICUs (8), no info (5)). Of the entire panel, 16 participants were either senior clinicians (3), directors or heads of department (7) or professors (6, thereof 2 in nursing). More than half of the panelists (17) had > 10 years of work experience in a critical care environment, whereof 9 panelists have worked there even > 20 years (further demographic details and graphical illustration in online supplements c, figs. S2, S3, S4 and S5). Participation with completion of the entire survey per round ranged from 78–92% completion rate over the five rounds of voting (R1: 92%, R2: 85%, R3: 78%, R4: 85%, R5: 85%; for further details see online supplements c, tab. S2). The online Delphi process was conducted between June 24^th^, 2020 and March 21^st^ 2021. Results per round are shown in Fig. 2.

The detailed results per round with voting split, provided and edited comments are given in online supplements c with results description per round (doc.3) and results tables per round (doc. 4, tab. S3: results round 1, tab. S4: results round 2, tab. S5: results round 3, tab. S6: results round 4, tab. S7: results round 5, tab. S8: results round 5, tab. S9: phrasing comparison round 5 and final list, tab. S10: final ICU discharge criteria list). And the end of the Delphi process, 28 ICU discharge criteria reached final consensus, thereof 23 criteria reached consensus ≥ 90% and 5 criteria reached consensus of 75 – 88% (tab. S10). For 5 criteria a deletion from the list was agreed with ≥ 92% consensus, because these criteria became obsolete as aspects were finally covered through other rephrased criteria (tab. S8). Based on comments received in the 5^th^ round and discussion among the team of investigators, 8 criteria got changed in phrasing for simplification for the final criterial list although they have all met already the consensus threshold (tab. S9). The set of proposed ICU discharge criteria for adult patients in any type of ICU covers patient-specific as well as organization-specific discharge criteria to evaluate discharge readiness of the individual patient but also to assess organizational capabilities to allow patient discharge to the next lower level of care (general ward in this study context). 24 patient-specific criteria reflect on the different organ systems (respiratory system, cardiovascular system, central nervous system, urogenital system), pain, fluid loss and drainages, medication and nutrition, patient diagnosis, prognosis, and patient preferences. 8 out of the 24 patient-specific criteria evaluate as part of their criteria phrasing whether currently available capacities and competencies as well as available technical infrastructure at the receiving unit are in place to safely discharge the patient. 4 criteria focus on the institution specific boundary conditions that allow or don’t allow patient discharge (institution’s specific admission criteria of receiving unit, safety standards such as isolation or support measures for out of hours discharges, and current capacity, acuity, and workload levels at the receiving unit). For every criterion a binary decision metric was defined with values for “Fit for discharge” and “Needs further intensive care therapy / monitoring”*.* For consistency and ease of use in daily clinical practice, all criteria have been phrased in a way that they can be answered with the values “yes” or “no” in the binary decision metric. For 6 criteria concerning the patient’s vital parameters, a value calculation method has been defined: For “blood oxygenation”, the worst value must be above the defined threshold and the trend must be stable over a defined time frame. For “respiratory rate”, “heart rate” and “mean arterial pressure”, the worst value must be within the acceptable range and the trend must be stable over a defined time frame. For “cardiac rhythm” and “hemoglobin” the trend must be stable over a defined time frame. For a definition of an appropriate value evaluation time frame per criterion that indicates stability, thus preventing patient readmission within 48 h after ICU discharge, agreement within one of the proposed time frame categories was low. 42% has been reached as a maximum agreement level in only one criterion, followed by 2 criteria reaching 38% and 8 criteria reaching 33% agreement in one category. All other criteria reached lower agreement levels per proposed evaluation time frame category. Based on the heterogeneity of the answers, the investigators decided to take this aspect out from any further voting round as they didn’t expect reaching a significantly higher agreement level within the following two rounds. As a result, no specific criterion evaluation time frames can be proposed. However, as the phrasing of the value calculation method refers to a defined time frame, for implementation in daily clinical routine, this time frame needs to be specified once by the clinical decision makers and in context of the institution specific workflows of vital parameter measurement, documentation, and visualization. The votes distribution per criterion could serve as a first orientation.

Also, the question in the fourth round, who of the stakeholder group can evaluate best if the patient meets a criterion, resulted in very heterogenous results. Only for 9 out of 33 criteria an agreement of ≥ 70% was reached on one stakeholder. A general insight from the panelists’ comments and the voting distribution was, that a lot of criteria need to be evaluated by an interdisciplinary team, that often should even involve a clinician or nurse from the receiving unit. In the performed survey, there was no selection option for the interdisciplinary team. That is why panelists commented via the comments field on the need to have an interdisciplinary team (13 related comments). Based on these results, it was decided to not further iterate on the decision maker. The investigators would rather recommend for criteria implementation in daily clinical practice to define the best decision maker(s) per criterion based on institution specific roles and collaboration aspects. Based on the comments, for some criteria certain roles can equally assess discharge readiness, often depending more on competency and experience level than on the specific role. An interdisciplinary team including representatives from the receiving unit should be consulted for criteria that reflect on patient specific criteria in context with capabilities of the receiving unit. For clinical practice implementation, it is also important to know the importance rank of each criterion, meaning if it is mandatory to meet the criterion or not and if there are exceptions in place that allow to overrule the criterion. After some simplification of the ranking, 17 out of 28 criteria reached > 90% consensus on “mandatory to be met”. Eleven criteria reached 73%—89% consensus on “mandatory to be met”. End-of-life care and agreed treatment limits where the exceptions mentioned the most (in 12 criteria), when “mandatory to be met” can be overruled. Looking at the voting alternatives, the highest vote for “not mandatory to be met” with 26% was reached for the criterion “Patient’s preference is to stop intensive care therapy and to leave the ICU”.

Finally, over a course of 5 rounds of voting on a basis of 40 initial ICU discharge criteria, where several criteria have been added, edited and deleted along the way through the panel’s consensus, the study resulted in 28 in-depth defined ICU discharge criteria for adult patients, that should be applicable to any type of ICU (Table 1).

**Table 1:** Final list of consented ICU discharge criteria

	**Final list of ICU discharge criteria**	**Fit for ICU discharge**	**Needs further intensive care therapy / monitoring**	**Value calculation method to evaluate discharge readiness**	**Criterion importance rank**	**Exceptions when "mandatory to be met"**
Respiratory system						
1	Is the patient's (own / artificial) airway patent?	yes	no	n.a	mandatory to be met	EOL-care, agreed treatment limits
2	Is the cough effective in a way, that the patient can be handled at the receiving unit?	yes	no	n.a	mandatory to be met	EOL-care, agreed treatment limits, p. with low level of consciousness and requiring long-term artificial airway aspiration
3	Blood oxygenation: Stable SpO2 (≥ 92% OR stable around lower patient individual baseline value) AND patient is breathing on room air?	yes	no	Worst value must be above threshold value AND trend must be stable over defined time frame	mandatory to be met	EOL-care, agreed treatment limits, p. with chronic lung disease
4	Respiratory rate: Stable RR trend with 10 ≤ resp ≤ 30 (pm) OR patient's individual baseline value is met?	yes	no	Worst value must be within acceptable range AND trend must be stable over defined time frame	mandatory to be met	EOL-care
5	If further respiratory support is needed, feasible at the receiving unit?	yes	no	n.a	mandatory to be met	
6	If the patient is in need of tracheal suctioning, feasible at the receiving unit?	yes	no	n.a	mandatory to be met	
7	If the patient is in need of long-term tracheostomy, could adequate care be provided at the receiving unit?	yes	no	n.a	mandatory to be met	If patient is sufficiently trained and capable to take care of the tracheostomy
Cardiovascular system						
8	Heart rate: Stable HR trend with 50 ≤ hr ≤ 110 (bpm) OR patient's individual baseline value is met?	yes	no	Worst value must be within acceptable range AND trend must be stable over defined time frame	mandatory to be met	EOL-care, agreed treatment limits, pre-existing bradycardia
9	Cardiac rhythm: Stable cardiac rhythm OR tolerable intermittent arrhythmia over defined time frame?	yes	no	Trend must be stable over defined time frame	mandatory to be met	EOL-care, agreed treatment limits, p. has intermittent AF but is otherwise hemodynamically stable with controlled ventricular response
10	Mean arterial pressure: Stable MAP trend with 60 < map ≤ 110 (mmHg) OR patient's individual baseline value is met?	yes	no	Worst value must be within acceptable range AND trend must be stable over defined time frame	mandatory to be met	EOL-care, agreed treatment limits
11	Hypervolemia / hypovolemia: Does the current volemia status require ICU monitoring?	no	yes	n.a	mandatory to be met	
12	Hemoglobin value stable over defined time frame?	yes	no	Trend must be stable over defined time frame	mandatory to be met	EOL-care, agreed treatment limits
13	Significant active bleeding or high risk of significant bleeding?	no	yes	n.a	mandatory to be met	EOL-care, agreed treatment limits
14	If the patient needs continued monitoring at the receiving unit, are required technology / staff capabilities in place?	yes	no	n.a	mandatory to be met	
15	If the patient carries a percutaneous transient pacemaker, could that be handled at the receiving unit?	yes	no	n.a	mandatory to be met	
16	If low-dose vasoactives are in use, is the patient manageable at the receiving unit?	yes	no	n.a	mandatory to be met	
Central nervous system						
17	Can the neurological status of the patient be adequately handled and monitored at the receiving unit?	yes	no	n.a	mandatory to be met	
Pain						
18	Pain therapy sufficient and feasible at the receiving unit?	yes	no	n.a	mandatory to be met	
Urogenital system						
19	Do urine output, electrolyte level, and renal function allow patient discharge?	yes	no	n.a	mandatory to be met	EOL-care, agreed treatment limits
20	If required, renal replacement therapy possible outside the ICU?	yes	no	n.a	mandatory to be met	EOL-care, agreed treatment limits
Fluid loss and drainages						
21	Could fluid loss or drainage(s) be monitored and handled adequately at the receiving unit?	yes	no	n.a	mandatory to be met	
Medication and nutrition						
22	If the patient needs continuous IV application (e.g. Insulin, glucose, antibiotics, vasopressors, nutrition), allowed and feasible at the receiving unit?	yes	no	n.a	mandatory to be met	
Patient diagnosis, prognosis and preferences						
23	Patient's preference is to stop intensive care therapy and to leave the ICU	yes	no	n.a	not mandatory to be met	
24	Therapeutic susceptibility: Patient doesn't benefit from ICU care anymore and negative effects may outweigh	yes	no	n.a	mandatory to be met	
Institution specific criteria						
25	Patient no longer meets ICU admission criteria and meets admission criteria for a lower level of care	yes	no	n.a	mandatory to be met	
26	Do current acuity and dependency levels and current workload at the receiving unit allow to admit and take care of this patient?	yes	no	n.a	mandatory to be met	
27	If the patient is immune-compromised or infectious, could the patient be handled and adequately cared for at the receiving unit?	yes	no	n.a	mandatory to be met	
28	If discharge at night or weekend can't be avoided, are measures in place to protect patient safety?	yes	no	n.a	mandatory to be met	EOL-care, agreed treatment limits, priotarisation against patients with greater need for intensive care

## Discussion

The objective of this Delphi study to reach consensus on a standardized set of ICU discharge criteria has been achieved. In the voting process, we involved 28 clinicians and nurses with a dedicated expertise and research interest in ICU transfer processes, coming from divers geographical and professional backgrounds. That allowed us to get a comprehensive evaluation of the provided first proposal of ICU discharge criteria that we derived from our previous literature research.

From the beginning, patient as well as process-related conditions were in focus of the consensus process. As phrased in earlier studies, only the two perspectives together could give a holistic view on discharge readiness for an individual patient to a particular next lower level of care [5, 9, 12]. Patient-specific criteria should indicate a stable state of the patient for at least the next 48 h that allows safe discharge with a minimized readmission risk [24, 25]. Organization-specific aspects affect the quality and success of care transitions from an ICU to a lower level of care and should reflect on current ICU capacity strain driven by census level and nursing workload as well as the capabilities of the next lower level of care, communication and handover practices between departments, as well as an early definition of patient-centric care goals that are aligned throughout the patient pathway.

The first round of voting was started with two blocks of criteria: the patient-specific block with 30 criteria and the organization-specific block with 10 criteria. The study was successfully ended after the 5th round with 24 patient-specific and 4 organization-specific criteria. Patient-specific criteria were represented by a holistic view on the different organ systems and therapeutic interventions as well as patient’s autonomy, continuous care needs, the patient’s wish, and therapeutic susceptibility. Organization-specific criteria were aligned institution specific admission and discharge criteria, discharge timing and accompanying safety measures, available technology, and care capacities at the next lower level of care. Throughout the various rounds of voting, it became clear that patient- and organization-specific discharge readiness is deeply intertwined as among the patient-specific criteria, 12 criteria also reflect on the capabilities of the receiving unit in the way they were finally phrased.

### Multi-parameter and multi-stakeholder approach

With its multi-parameter and multi-stakeholder approach, this study uniquely corresponds to the request from several publications [5, 26–29], that ICU discharge criteria should not only determine when a patient is no longer in need of intensive care but also whether the receiving unit is capable to take appropriate care of that particular patient. Here, the checklist format could serve as a structured decision support tool to evaluate both perspectives and to facilitate a handshake on patient transition between the sending and receiving unit, thus improving current discharge practices. The high completion rate through all five rounds supports robust results and reflects the interest and dedication of the panelists for this area of research. Throughout the voting process, the panelists’ comments, and perspectives, based on their diverse geographical and professional background, lead to a comprehensive result. We purposely involved ICU clinicians and critical care nurses in the definition of ICU discharge criteria as the formalization of multidisciplinary input in the ICU discharge decision-making has been recommended in earlier work [30]. Research has also repeatedly shown that bedside nurses can offer a unique perspective on the type and amount of nursing care each patient needs, and nurse-physician collaboration in decision-making at the time of ICU discharge is associated with better patient outcomes, reduction in ICU readmission and hospital mortality [30–32]. Those studies also proposed that discharge readiness evaluation needs to consider nursing workload related criteria at the discharging and at the receiving unit, as well as available skill sets and patient-nurse ratios. Surprisingly, none of the proposed scores or ratios reflecting on nursing workload or patient-nurse ratio were finally consented. One criterion was formulated rather generic without any patient-nurse ratio, acuity level or workload scores, but specifically to the care capacities at the receiving unit (“Do current acuity and dependency levels and current workload at the receiving unit allow to admit and take care of this patient?”). This particular criterion would help to facilitate a discussion and ultimate handshake for the care transition but leave it still to the decision-making subjectivity and argumentation skills of the different stakeholders. For many of the other criteria in the final list, it was reflected per criterion if the patient status or the needed continuous interventions could be managed at the receiving unit. Having such a focus on the organizational aspects of discharge readiness, where different units need to evaluate if the discharge criteria are met, also brings the challenge of structuring access to the clinical decision support tool (“Who ticks the box?”) and organizing information flow around it (“Who gets notified?”). The questions, who can best evaluate the particular discharge criterion and who ultimately takes the discharge decision, leads to another remarkable result of our study: No clear preference for a particular decision maker per criterion and several comments to better have an interdisciplinary team deciding whether the criteria are met, also supports a multi-stakeholder approach in clinical decision making. Retrospectively, there should have been a selection option in the questionnaire for “interdisciplinary team”, which wasn’t there but would have potentially brought even clearer results for the multi-stakeholder approach. Another insight was, that clinicians and nurses from the general ward environment should have been included in the panel. Especially, as so many criteria were reflecting on the capabilities of the receiving unit, we assume that their input and vote would have brought an even more comprehensive result. With that insight, we recommend clinical implementation and validation of the criteria being undertaken by a multidisciplinary and interdepartmental team.

### High consensus level with the focus on generally available and applicable criteria

This study results could serve as a starting point for implementation in daily clinical practice in many European healthcare systems. However, the panelists represented with their expertise and input mainly very developed countries, where a high standard of intensive care is provided, although the availability of ICU beds per 100.000 capita of population varies widely [33]. The definition of a rather high consensus level with ≥ 90% for criteria inclusion and 5 rounds of partially reiterations of voting enabled consistency checks and fine-tuning of the proposed criteria. Throughout the different rounds of voting, the panelists were focused on the aim that the criteria should be widely available in clinical practice and as broadly applicable as possible, concerning the patient group, type of illness and type of ICU. Further, the inclusion of criteria around patient wishes, prognosis and therapeutic susceptibility, as well as the criteria importance ranking including the exceptions in case of a palliative care pathway, builds a standardized form that was asked for in earlier studies to evaluate the adequacy of the current treatment with the team, the patient and his relatives [34]. However, the results of the criteria importance ranking were quite heterogenous and therefore should serve as a first orientation whether some criteria are more important to be met than others. Implementation in clinical routine needs to show how useful the criteria importance ranking is. Capturing retrospectively how often a “mandatory to be met” criteria was actually not met when a patient was discharged and combining that with patient outcome data will bring more insights into how to apply this rule and how to further fine-tune the weighting of the criteria against each other or even combining some.

### Aspects for implementation in daily clinical practice

A possible operationalization of the criteria list could be in a kind of dashboard view in the Patient Data Management System (PDMS), where discharge readiness status is visualized as a summary visual per patient. There, it could automatically flag patients that are “fit for discharge”, when the required criteria thresholds are met over the institution-specific defined time frames. That would help the care team to quickly assess current capacity requirements when they are asked to admit a new patient. Furthermore, in a single patient view, the different factors impacting discharge readiness can be reviewed in more detail and current discharge barriers can be depicted. For hospitals still documenting in paper-based formats, a color-coded discharge readiness checklist could support individual patient assessment. This perspective could guide morning rounds to focus the attention to the most likely-dischargeable patients and on given discharge barriers. Further, the variety in clinical decision making could be reduced through guiding especially junior clinicians through a structured decision criteria catalogue. Implementing this list in daily clinical routine would help to drive multidisciplinary discussions around care goals, early consideration of alternative care pathways and, with that, adapting the discharge criteria to the patient-individual treatment plan. A continuous visualization of the discharge readiness assessment could prompt clinicians to consider patient discharge outside the morning rounds and throughout the day and help them plan and facilitate the actual patient transition with the related care team. Discharging patients as soon as they are stable enough would have great potential to optimize the use of limited ICU resources, to reduce waste in terms of overtreatment, waiting and avoidable complications and to reduce costs [2, 10].

### Criteria reporting automation

With 28 criteria, we still provide a rather long list of discharge criteria that need to be checked by the decision makers in a recurring manner to determine discharge readiness. Former publications also formulated the need that ideally, most of the defined criteria should be auto-fillable with data from PDMS and Electronic Medical Record (EMR) systems [12]. Although throughout the different rounds of voting, criteria were in discussion that could have been very well derived in electronic and automated form from the PDMS and EMR systems, most of those criteria didn’t make it to the final list. Remarkably, none of the proposed numeric scores and scales (from initial proposal and proposed by the panelists throughout the voting process), like Glasgow Coma Scale (GCS), Richmond Agitation Sedation Scale (RASS) and Confusion Assessment Method for Intensive Care Units (CAM-ICU), Sequential Organ Failure Assessment (SOFA) and delta SOFA, Pain and Frailty scales as well as nursing workload related scores reached final consensus. For those criteria on the final list, that require a calculation method to determine whether the criterion is within the threshold values (SpO2, respiratory rate (RR), heart rate (HR), cardiac rhythm, mean arterial pressure (MAP), hemoglobin (Hb)), a calculation and reporting automation connected with the criteria catalogue is critical for implementation success in daily clinical practice.

### Continuous discharge readiness evaluation

With the resulting list of discharge criteria, the request for a continuous evaluation of discharge readiness [12] can still not be met. Ultimately, only 6 criteria are truly continuously and automatically reportable. On the other hand, some of the criteria that are closely related to treatment capabilities or needed infrastructure at the receiving unit, only need to be checked once for a particular patient or don’t account for a particular receiving unit or institution specific workflow and could therefore be excluded when applied in that environment. For the remaining criteria it may help to define evaluation time frames on an institution level. That means, over which time frame a certain criterion needs to be met to indicate patient stability. The results from the 4th round on suitable evaluation time frames didn’t show strong preferences for particular evaluation time frames per criterion. Within the investigators team, it was suspected that also further iterations on this question wouldn’t bring any significantly clearer results. For the sake of survey simplification, it was decided to stop the query on the criteria evaluation time frames after only one round of results. However, the vote distribution on proposed criteria evaluation time frames could serve as a first orientation to define institution specific time frames that match the related workflows, and to trigger future research on this aspect. Further, for daily practice implementation in PDMS- as well as paper-based ICUs, it remains a challenge to provide the basis for a discharge readiness evaluation possible at any time. Different types of data and resources need to be linked to provide one comprehensive view for all involved stakeholders on the patient’s and organization’s progress towards discharge readiness, decisions taken and access to underlying data.

### Need for clinical implementation research

A multicenter point-prevalence study could compare actual discharge decision making criteria against the consented list and illustrate current differences in discharge practices. Implementation studies should further demonstrate if this consented standardized set of discharge criteria can adequately assess ICU patient’s discharge readiness, by reviewing fit for discharge status, patient flow, capacity utilization and patient outcomes key performance indicators (KPIs), and comparing them to baseline measurements from common clinical practice. A defined and clinically validated Fit for discharge-status could help future root cause analysis to identify discharge barriers and to measure process related waste of ICU capacities. Ultimately, it needs to be researched in how far the implementation of objective and standardized ICU discharge criteria can reduce waste in the ICU discharge process, increase overall ICU capacity utilization and workflow efficiency. Clinical practice implementation may also stimulate future research on how this set of discharge criteria can further be improved towards an automated and intelligent clinical decision support tool, suitable to integrate aggregated data in form of scores and ratios, see trends, predict patient individual discharge readiness, and learn retrospectively about factors that determine successful patient discharge and pathway selection.

### Strengths and limitations

This study has several strengths. The expert panel included a decent number of subject matter experts in the field of acute care transitions, coming from different professional and geographical backgrounds, so that the results of the study should be robust against regional practice differences. The group was also not too large, so that the high proportion of provided qualitative answers could be handled well. Anonymity of the experts and their individual responses were preserved throughout the entire Delphi process, to avoid bias due to individual’s dominance and group pressure. Several iteration steps and related fine-tuning, the high completion rate throughout the five rounds of voting as well as the high level of consensus all helped to build a robust and consistent result.

However, our work has certain limitations. Our panel was limited to ICU clinicians and ICU nurses from different healthcare systems, types of hospitals and levels of work experience. It didn’t include other stakeholders in the ICU discharge readiness evaluation process, such as receiving units’ clinicians and nurses, or the experiences of patients and rapid response teams. Including those parties and aspects in future research in this context would make the results more robust and also may increase acceptance when implemented in clinical practice. Further, having a multinational panel answering an online questionnaire in English language with no possibility to clarify doubts could lead to misinterpretation of questions or also provided statements. Some comments were provided in national language and needed to be translated. Also, in round 1 and 2, a few experts were confronted with criteria, in particular different types of nursing workload scores, they had no experience with in their own clinical practice. So, their ability to judge on its general usability could be limited. It could be questioned, if that had an effect that those criteria didn’t receive sufficient consensus and got excluded after round 2. Further, the inclusion of the multidisciplinary team in the 4^th^ and 5^th^ round, could have brought a stronger agreement on a potential decision maker per criterion. But the need for this was only revealed through the provided comments. Also, a reiteration on the criteria evaluation time frames in further rounds could have brought clearer results, that could be then linked to the value calculation method for clinical practice implementation. This missing link requires further research on this detail. In general, several concerns around clinical practice implementation have been raised via the panelists comments and have been summarized in the discussion part and translated into suggestions for future implementation research.

## Conclusion

In daily clinical practice, there is an absence of evidence-based and well-defined ICU discharge criteria that reflect a holistic assessment on the patient’s fit for discharge status as well as on the organizational capabilities that allow a safe and timely transition to the next lower level of care. A critical care expert panel consented via a modified online Delphi process on a final list of 28 criteria to evaluate discharge readiness in adult ICU patients for a care transition to a general ward environment. The set of criteria covers patient-specific aspects, such as a holistic view on organ systems, therapeutic interventions as well as patient’s autonomy, continuous care needs, patient’s preferences, and therapeutic susceptibility. The consented organization-specific criteria focus on the underlying framework conditions, like discharge timing, safety measures, available care capacities, skill sets and technology. First clinical practice implementation studies are recommended to further define criteria evaluation time frames, the role of the different stakeholders in the decision process and the criteria importance ranking. Future research shall focus on validation of the criteria set, utility, criteria reporting and decision support automation, and visualization.

In a broader perspective, applying clearly defined ICU discharge criteria may reduce decision making subjectivity, improve patient safety and workflows in daily care transitions, support efficient use of limited ICU resources and equity of care, but also prevent avoidable patient deterioration and overburdening of lower levels of care. That means, patients and organizations could benefit from the implementation of such discharge criteria as a clinical decision support in daily clinical practice.

## Supplementary Information

**Additional file 1.**

**Additional file 2.**

**Additional file 3.**

**Additional file 4.**

## Data Availability

The raw datasets used and/or analyzed during the current study are not publicly available due to the provided detailed comments in different languages that could impair anonymity of the rather small expert panel. An English language high-level summary of all comments per round, that served as further decision base to set up the following round, is provided in the online supplement c, doc. 3 and 4. Raw data sets can be made available in an anonymous form from the corresponding author on reasonable request.

## Abbreviations

ICU: Intensive Care Unit
LOS: Length of stay
ED: Emergency department
PDMS: Patient Data Management System
EMR: Electronic Medical Record
GCS: Glasgow Coma Scale
RASS: Richmond Agitation Sedation Scale
CAM-ICU: Confusion Assessment Method for Intensive Care Unit
SOFA: Sequential Organ Failure Assessment
RR: Respiratory Rate
HR: Heart Rate
MAP: Mean Arterial Pressure
Hb: Hemoglobin
KPI: Key Performance Indicator
