# Supplementary material for: Delphi study to derive expert consensus on a set of criteria to evaluate discharge readiness for adult ICU patients to be discharged to a general ward—European perspective
Source: BMC Health Serv Res. 2022 Jun 13;22:773. doi: 10.1186/s12913-022-08160-6 (PMC9190161; doi:10.1186/s12913-022-08160-6)
Supplement: Supplementary file 1 — Additional file 1. [file 12913_2022_8160_MOESM1_ESM.docx]

**Delphi study to derive expert consensus on a set of criteria to evaluate discharge readiness**

**for adult ICU patients to be discharged to a general ward**

**- European perspective**

**Pre-read document for invited experts to a Delphi study to reach consensus on a set of discharge criteria applicable in adult ICUs**

Maike Hiller MA^1,4^, Maria Wittmann, MD^2^, Hendrik Bracht MD PhD^3^, Jan Bakker MD PhD FCCM FCCP ^1,5,6^

1 Erasmus MC University Medical Center, Dept. of Intensive Care Adults Rotterdam, The Netherlands

2 University Hospital Bonn, Dept. of Anesthesiology and Intensive Care Medicine, Bonn, Germany

3 Central Emergency Medicine Services and Department of Anesthesiology and Intensive Care Medicine, University Hospital Ulm, Ulm, Germany

4 Philips Medizin Systeme Böblingen, Dept. of Monitoring and Analytics, Clinical Services, Böblingen, Germany

5 New York University School of Medicine and Columbia University College of Physicians & Surgeons, New York, USA

6 Pontificia Universidad Catolica de Chile, Dept of Intensive Care. Santiago, Chile

**The broader objective: Optimizing use of ICU resources by proactively managing the ICU patient discharge process to the next lower level of care**

Intensive care capacities are scarce and costly resources. Increasing the number of ICU beds is often not an option due to financial limitations in the healthcare system and even more often due the lack of specialized and highly skilled care givers to staff the beds. Facing these limitations, any measure to optimize the utilization of ICU resources is a high priority for hospital management. In a broader context, the ICU stay is an element of a patient’s pathway through the hospital. Therefore, focusing on optimal patient outflow of the ICU can positively impact the performance of the admitting departments (1-3). Timely admissions of critical patients, reduction of any type of wasted procedures during ICU stay and a standardized and safe discharge process that limits readmission rates, enables a positive patient outflow of the ICU (4-7). Ideally, patients in the ICU should be continuously evaluated to identify those who no longer require ICU treatment and are fit enough to be transferred to the next care level. Discharging the right patient at the right time reduces LOS, readmission rates, and costs, where an inappropriate discharge will achieve the opposite and increase risk of mortality (8, 9). There are however many factors that affect this process.

First, ICU census has an impact on patient flow and discharge timing. Current high ICU census sometimes forces care providers to discharge patients earlier in order to free up beds for patients that require intensive care at priority. However, premature discharge may lead to adverse events followed by readmission to the ICU (10). That in return will even increase the capacity strain at the ICU, overall LOS, risk of mortality and costs (6, 10). Second, the availability of step-down units or ward beds and dedicated care capacities are important for a positive patient outflow (9, 11, 12) but they are often only available in larger hospitals. In contrast, overestimation of care capacities at the receiving ward may lead to adverse events and readmission to the ICU (13). Third, lack of communication and handover practices between departments, such as ICU and general ward, may slow down timely discharge and negatively impact patient safety. In return, research has shown that implementing best practice handover interventions has a positive effect on the quality of patient handover and flow (13). Fourth, unspecific criteria and lack of processes to determine therapeutic susceptibility, as well as late consideration of patient's wishes and alternative care pathways may lead to ICU discharge delay, excess burdens and increased costs of care. Therefore, the adequacy of treatment with respect to therapeutic susceptibility and patient wishes needs to be regularly evaluated with the team, the patient and his family (5). Finally, the absence of standardized discharge decision metrics and formal guidance, and the lack of aggregated data to enable objective judgement on individual patient’s risk brings a variety in clinical decision making. Differing decisions are strongly related to the clinicians’ experience and comfort level and whether structural and organizational framework conditions are reflected (3, 14, 15). To improve patient safety and workflow efficiency, patient- as well as process-related conditions should be formulated as specific discharge parameters, embedded in a standardized discharge and handover process (13, 16).

The need for objective and widely applicable criteria to evaluate patient discharge readiness is there for clear and has been voiced by a variety of studies (3, 13, 14, 16, 17). Therefore, the motivation behind this study is to come up with a comprehensive proposal of discharge criteria for adult ICUs that is widely applicable in daily clinical practice throughout Europe~~.~~ The set of criteria for an objective evaluation of patient discharge readiness should satisfy two purposes: First, patient specific criteria should indicate a stable state of the patient for at least the next 48 hours that allows safe discharge with a minimized risk of readmission. Second, the set of criteria should incorporate system-specific criteria that would allow comparison across units to identify suboptimal use of resources and help to reduce waste of ICU capacities. As a basis, we conducted a scoping literature review focusing on studies where ICU discharge practice optimization was in focus. The limited number of proposed discharge criteria and best practice examples in European ICUs were reviewed and evaluated with respect to the above-mentioned factors that affect the efficiency of the discharge process. The evaluated criteria and recommendations have been condensed, structured and referenced in the table at the end of this document. This table of criteria will be subject to a Delphi study, to reach consensus on a standard set of discharge criteria, widely applicable for adult patients in European intensive care settings. Implementing those in daily clinical practice should help to guarantee equity in care provision, establish positive patient flow and improve the quality of care transitions in the interest of the patient and the society (15).

**Which criteria are suitable to have an objective and widely applicable evaluation framework on individual patient’s discharge readiness in adult ICU environments?**

The patient related criteria in the table consider patient status, interventions and medications, diagnosis and prognosis as well as patient’s preferences that could support or contraindicate discharge readiness. Further, it includes nursing workload related criteria as well as institutional factors. The aim of this list of discharge criteria is to support a holistic view on patient discharge readiness and increase objectivity and comparability in clinical decision making.

Beside the previous aspects, implementation in daily clinical practice should be in focus when defining a set of widely applicable ICU discharge criteria. Ideally, most of the defined criteria should be auto-fillable with data from PDMS and EMR systems. Further, the criteria that go into the standard set of discharge criteria should be widely available in clinical practice, suitable to see trends and predict discharge readiness, modifiable with regards to different patient pathways and should include a multi-stakeholder’s view on discharge readiness.

These requirements are supported by one of the future directions of the Admission, Discharge and Triage guidelines by Nates et al. They suggested to utilize electronic medical record documentation to automatically capture patient characteristics and to develop a self-populating tool for ADT-decisions that supports determining patient placement (16). A possible realization of this direction could be a kind of dashboard view in the PDMS where discharge readiness status is visualized per patient. There, it could automatically flag patients that appear fit for discharge. That would help the care team to quickly assess capacity requirements when they are asked to admit a new patient. Furthermore, in a single patient view, the different factors impacting discharge readiness can be reviewed in more detail displaying the underlying information. For hospitals still documenting in paper-based formats, a color-coded discharge readiness checklist could support individual patient assessment. This perspective could guide morning rounds to focus the attention to the most likely-dischargeable patients. In addition, it could also support bedside discussions around goals of care, and the planning and facilitation of the actual patient discharge. Having this information continuously available, could prompt clinicians to consider patient discharge outside the morning rounds and throughout the day. Discharging patients as soon as they are stable enough would have great potential to optimize use of ICU resources, to reduce waste in terms of overtreatment, waiting and avoidable complications and to reduce costs (3, 18).

With that perspective in mind, we would like to ask you to participate in the Delphi approach, to be conducted as follows.

**The Delphi study**

The objective of the Delphi study is to reach expert consensus on a European level on a standardized set of discharge criteria. The set of discharge criteria shall be suitable to comprehensively evaluate discharge readiness of individual patients by clinicians and nurses. Objective decision support shall enable discharges to be more timely, safer and oriented on patient severity of illness and organizational capabilities.

The Delphi study is conducted in three stages:

1^st^ stage: Preselection of criteria based on literature review.

The proposed list was selected by the investigators based on an extensive literature review and it was reviewed by selected experts for comprehensiveness. Items, values and ranges were added or edited where applicable.

2^nd^ stage: Online-Delphi process

By using an electronic voting platform designed for Delphi studies (welphi.com), the group of experts participating in this process votes on every criterion for inclusion in the set of discharge criteria. The aim is to reach consensus on the set of discharge criteria that is able to evaluate individual patient’s discharge readiness for adult patients in any type of intensive care unit and not specific to any individual disease process or specialty. Consensus will be achieved through five rounds of voting, one open and four closed Delphi rounds, via online surveys.

Any criteria where a consensual agreement of ≥ 90% will be reached, will automatically be included in the final set. Criteria with consensual agreement between 75% and 89% will be refined in the next rounds of voting. Any criteria with a consensus < 75% will be excluded (19).

1^st^ round - Open Delphi round:

Each expert will review each criterion on the proposed list. Per criterion the options are:

- “Don’t do anything”, then the expert agrees that the criterion will go to the next round.
- “Comment on needed changes”, then the investigators will review the change request entered by the expert and adapt the criterion or phrasing if needed for the next round.
- “Comment with ‘remove’” in case the expert feels the criterion is not at all relevant. Then the investigators will remove the criterion for the next round if ≥ 25% of the experts voted for removal.
- “Add a criterion to the list”, if the expert thinks, there is a relevant criterion missing in the proposed list. This criterion will then be included in the next round.

2^nd^ round – Closed Delphi rounds:

The Delphi group will receive the results of the open Delphi round. Each expert ranks each criterion on the list using a 5-point Likert scale (very relevant, relevant, cannot judge, not relevant, completely irrelevant). Positive inclusion of a criterion at this stage will be defined as answering either very relevant or relevant to the question. Criteria reaching ≥ 90% agreement will automatically be included in the final set. Criteria reaching < 75% agreement will be automatically excluded. Criteria reaching 75% - 89% agreement will go to the next round.

3^rd^ round:

The Delphi group will receive the results of the 2^nd^ round and outliers will be asked to make a statement if they change their opinion towards the group’s opinion or if they stay with their judgement. Out of the results of the 3^rd^ round plus the criteria with ≥ 90% agreement from the 2^nd^ round, a final criteria list will be defined by taking all criteria with a consensus of ≥ 90%.

4^th^ round:

The Delphi group will receive the final list of criteria and will be asked to define the importance of each criterion (individual criterion not met already prohibits discharge vs good if it is met or irrelevant in case of discharge to palliative care**)**, to define the time window throughout that a certain criterion needs to be met, to agree with the proposed value or propose a different value and the way how the value should be calculated.

5^th^ round:

Results will be reported back to the Delphi group in form of median or other type of result summary. The kind of result summary, illustration and consensus targets per criterion and value will be defined by the investigators based on the received feedback from the 4^th^ round. For refinement, results will be reported back to outliers in order to ask them whether they want to adapt their answers to the majority or stay with their answer.

3^rd^ stage: Conclusion on final results

The final results will be shared with the expert group.

**Tab. S1 Initial proposal of potential ICU discharge criteria as a basis for the Delphi process**

| **Discharge readiness evaluation criteria** | | | | | | | | |
| --- | --- | --- | --- | --- | --- | --- | --- | --- |
| **Is patient fit for discharge? (patient-related factors)** | | | | | | | | |
| **Patient status** | | | | | | | | |
| **ID** | **List of criteria** | | **Criterion importance** | **Criterion evaluation time frame** | **Binary decision metric** | | **Reference** | **Value calculation** |
|  |  |  | **Selection options per criterion:**  **Individual criterion not met already prohibits discharge / good if it is met /**  **irrelevant in case of discharge to palliative care** | **For how many hours should each criteria value be met to indicate a stable state of the patient for at least the next 48 hours that allows safe discharge with a minimized risk of readmission?** | **Fit for discharge** | **Need further intensive care therapy/monitoring** | (3)  (11) | For defined time window, how should the value be calculated?  E.g. median, trend +/-%, worst value, last value, last score, patient baseline value, something else? |
|  |  | **Respiratory system** |  |  |  |  | (2),  (3),  (20)  (14) | n.a. |
| 1 |  | Airway patent |  | ≥ X hours | yes | no |  | n.a. |
| 2 |  | FiO2 |  | ≥ X hours | patient individual baseline level met | patient individual baseline value not met |  |  |
| 3 |  | Shall blood oxygenation be used for discharge readiness assessment? If yes, which method? |  |  |  |  |  |  |
| 3a |  | either Peripheral blood oxygenation |  | ≥ X hours | SpO2 ≥ 95(%) | SpO2 < 95 (%) |  |  |
| 3b |  | or Arterial oxygenation |  | ≥ X hours | SaO2 ≥ 95(%) | SaO2 < 95(%) |  |  |
| 4 |  | Respiratory rate |  | ≥ X hours | 10 ≤ resp ≤30 (pm) | resp < 10 or > 30 (pm) |  |  |
|  |  | **Cardiovascular system** |  |  |  |  | (2),  (3),  (20)  (14), (21), (22) | n.a. |
| 5 |  | Systolic blood pressure |  | ≥ X hours | bp ≥ 100 (mm Hg) | bp < 100 (mm Hg) |  |  |
| 6 |  | Mean arterial pressure |  | ≥ X hours | map > 65 mmHg | map ≤ 65 mmHg |  |  |
| 7 |  | Heart rate |  | ≥ X hours | hr 60 ≤ min ≤ 100 (bpm) | hr < 60 or > 100 (bpm) |  |  |
| 8 |  | Hypervolemia / hypovolemia |  | ≥ X hours | no | yes |  | n.a. |
| 9 |  | Hemoglobin value stable |  | ≥ X hours | yes | no |  | n.a. |
|  |  | **Pain** |  |  |  |  | (2),  (3)  (22) | n.a. |
| 10 |  | Do you use a pain scale for discharge readiness assessment? |  |  |  |  |  | n.a. |
| 10a |  | If yes, please name the scale below and indicated values that either allow or prohibit discharge: |  | ≥ X hours | Pain scale value??? | Pain scale value??? |  |  |
| 11 |  | Pain therapy sufficient and feasible at next lower level of care? |  | ≥ X hours | yes | no |  | n.a. |
|  |  | **Central nervous system** |  |  |  |  | (2)  (20) (3) (23)  (22) | n.a. |
| 12 |  | Hyperkinetic / Hypokinetic delirium: |  | ≥ X hours | no | yes |  | n.a. |
| 13 |  | Glasgow Coma Scale |  | ≥ X hours | gcs ≥ 14 | gcs < 14 |  |  |
| 14 |  | and / or Richmond Agitation-Sedation Scale (RASS) |  | ≥ X hours | RASS 0 - 1 | RASS < 0 or > 1 |  |  |
| 15 |  | and / or Confusion Assessment Method in Intensive Care (CAM-ICU) |  | ≥ X hours | CAM-ICU < 3 mistakes | CAM-ICU > 3 mistakes |  |  |
| 16 |  | and / or Intensive Care Delirium Screening Checklist (ICDSC) |  | ≥ X hours | ICDSC score < 4 | ICDSC ≥ 4 |  |  |
| 17 |  | Need for fixation due to risk potential of self or others endangerment? |  | ≥ X hours | no | yes |  | n.a. |
| 18 |  | **Temperature** |  | ≥ X hours | 36 ≤ temp ≤ 37.5 (°C) | temp < 36 (°C) or > 37.5 (°C) | (2) (3) (22) |  |
| 19 |  | **Gastrointestinal passage** |  | ≥ X hours | not blocked | blocked / requires therapy | (22) | n.a. |
| 20 |  | **Large fluid losses via drainage** |  | ≥ X hours | no | yes | (22) | n.a. |
| **Patient's diagnosis** | | | | | | | | |
| 21 |  | Chronic health and physiological reserve  (assessed through functional capacity, co-morbid disease, age) |  |  | allows discharge | doesn't allow discharge | (5), (20) | n.a. |
| 22 |  | Frailty scale:  Could patient’s frailty be handled at next lower level of care? Please review proposal and provide acceptable score. |  |  | yes  CFS 1-6, 9??? | no  CFS 7 -8 ??? | (24) |  |
| **Patient prognosis and preference** | | | | | | | | |
| 23 |  | Sabadell score from 0 (patients with expected good long-term prognosis) to 3 (patients not expected to survive the hospital stay) |  |  | –0-1 | 2-3 | (25) |  |
| 24 |  | Patient’s preference is to stop intensive care therapy and leave the ICU |  |  | yes | no | (5) | n.a. |
| 25 |  | **Therapeutic susceptibility:**  Patient doesn’t benefit from ICU care anymore, and negative effects may outweigh |  |  | yes | no | (5, 16, 26) | n.a. |
| **Patient related nursing workload** | | | | | | | | |
| 26 |  | Which of the indices below are relevant to assess patient’s discharge readiness?  Please indicate appropriate values for the relevant nursing workload indices that allow patient discharge to next lower level of care: |  |  |  |  |  | n.a. |
| 26a |  | NAS |  |  | ??? | ??? | (14) (27) (28) (29, 30) |  |
| 26b |  | TISS28 |  |  | ??? | ??? |  |  |
| 26c |  | SWIFT |  |  | ??? | ??? |  |  |
| 26d |  | PACS |  |  | ??? | ??? |  |  |
| 26e |  | Other, please name: |  |  | ??? | ??? |  |  |
| **Institution specific criteria determining the ultimate time and date of actual discharge**  **(process factors, depending on care facility set-up and care team capabilities, values are a proposal and should be modified per institutional policy)** | | | | | | | | |
| 27 |  | Patient no longer meets ICU admission criteria and meets admission criteria for a lower level of care. |  |  | yes | no | (16) | n.a. |
| 28 |  | Possibility to discharge patient at higher risk for mortality and readmission (high severity of illness, multiple comorbidities, physiologic instability, ongoing organ support) to a step-down unit or long-term acute care hospital |  |  | yes | no | (16) | n.a. |
| 29 |  | If discharge at night or weekend can't be avoided, are measures to protect patient safety available? |  |  | yes | no | (16); (31) (32) | n.a. |
| 30 |  | Renal replacement therapy possible outside ICU? |  |  | yes | no | (14) | n.a. |
| 31 |  | Wound dressing manageable at next lower level of care? |  |  | yes | no | (28) | n.a. |
| 32 |  | If further respiratory support needed, feasible at next lower level of care? |  |  | yes | no | (27, 33) | n.a. |
| 33 |  | If patient needs continuous insulin /glucose infusion, feasible at next lower level of care? |  |  | yes | no |  | n.a. |
| 34 |  | If patient is dependent on intravenous antibiotics, feasible at next lower level of care? |  |  | yes | no |  | n.a. |
| 35 |  | ICU outreach services in place (have ICU consultant available to facilitate transition from ICU, assist ward staff in the management of deteriorating patients, facilitate transfer to ICU if needed) |  |  | yes | no | (16) (33) | n.a. |
| 36 |  | Use of alternative care pathways e.g. palliative care, discharge reasons clearly documented, support plan in place for patient and family |  |  | yes | no | (5) | n.a. |

**References**

1. Bhattacharjee P, Kumar Ray P. Patient flow modelling and performance analysis of healthcare delivery processes in hospitals: A review and reflections. Computer & Industrial Engineering. 2014;78:299-312.

2. Knight G. Nurse-led discharge from high dependency unit. Nurs Crit Care. 2003;8(2):56 - 61.

3. McWilliams C, Lawson DJ, Santos-Rodriguez R, Gilchrist ID, Champneys A, Gould TH, et al. Towards a decision support tool for intensive care discharge: machine learning algorithm development using electronic healthcare data from MIMIC III and Bristol, UK. BMJ Open. 2019;9(e025925).

4. Levin PD, Worner TM, Sviri S, Goodman SV, Weiss YG, Einav S, et al. Intensive Care Outflow Limitation - Frequency, Etiology and Impact. Journal of Critical Care. 2003;18(4):206-11.

5. Bion J, Dennis A. ICU admission and discharge criteria. In: Webb A, Angus D, Finfer S, Gattinoni L, Singer M, editors. Oxford Textbook of Critical Care: Oxford University Press; 2016. p. 86-9.

6. Howell MD. Managing ICU throughput and understanding ICU census. Curr Opin Crit Care. 2011;17(6):626-33.

7. Howell MD, Stevens JP. Rationing without comtemplation: Why attention to patient flow is important and how to make it better. In: Scales DC, Rubenfeld GD, editors. The Organization of Critical Care. New York: Springer Science+Media New York; 2014. p. 155-75.

8. Wunsch H, Nguyen YL, Angus DC. Smoothing the way: Improving admission to and discharge from the ICU. In: Flaatten H, Moreno RP, Putensen C, Rhodes A, editors. Organization and Management of Intensive Care. Berlin: European Society of Intensive Care Medicine; 2010. p. 269 - 76.

9. Johnson DW, Schmidt UH, Bittner EA, Christensen B, Levi R, Pino RM. Delay of transfer from the intensive care unit: a prospective observational study of incidence, causes, and financial impact. Crit Care. 2013;17(4):R128.

10. Chan CW, Farias VF, Bambos N, Escobar GJ. Optimizing ICU discharge decisions with patient readmissions. Operations research. 2012;60(6):1323-41.

11. Kramer AA. A novel method using vital signs information for assistance in making a discharge decision from the intensive care unit. Medical Research Archives. 2017;5(12):1-12.

12. Iapichino G, Radrizzani D, Bertolini G, Ferla L, Pasetti G, Pezzi A, et al. Daily classification of the level of care. A method to describe clinical course of illness, use of resources and quality of intensive care assistance. Intensive Care Med. 2001;27:131-6.

13. van Sluisveld N, Oerlemans A, Westert G, van der Hoeven JG, Wollersheim H, Zegers M. Barriers and facilitators to improve safety and efficiency of the ICU discharge process: a mixed methods study. BMC Health Serv Res. 2017;17(1):251.

14. Heidegger CP, Treggiari MM, Romand JA, Swiss ICUN. A nationwide survey of intensive care unit discharge practices. Intensive Care Med. 2005;31(12):1676-82.

15. Capuzzo M, Moreno RP, Alvisi R. Admission and discharge of critically ill patients. Curr Opin Crit Care. 2010;16(5):499-504.

16. Nates JL, Nunnally M, Kleinpell R, Blosser S, Goldner J, Birriel B, et al. ICU Admission, Discharge, and Triage Guidelines: A Framework to Enhance Clinical Operations, Development of Institutional Policies, and Further Research. Crit Care Med. 2016;44(8):1553-602.

17. Stelfox HT, Lane D, Boyd JM, Taylor S, Perrier L, Straus S, et al. A scoping review of patient discharge from intensive care: opportunities and tools to improve care. Chest. 2015;147(2):317-27.

18. Almoosa KF, Luther K, Resar R, Patel B. Applying the New Institute for Healthcare Improvement Inpatient Waste Tool to Identify "Waste" in the Intensive Care Unit. J Healthc Qual. 2016;38(5):e29-38.

19. Rhodes A, Moreno RP, Azoulay E, Capuzzo M, Chiche JD, Eddleston J, et al. Prospectively defined indicators to improve the safety and quality of care for critically ill patients: a report from the Task Force on Safety and Quality of the European Society of Intensive Care Medicine (ESICM). Intensive Care Med. 2012;38(4):598-605.

20. Fakhry SM, Leon S, Derderian C, Harakeh H, Ferguson PL. ICU bounce back in trauma patients: An analysis of unplanned returns to the intensive care unit. J Traum Acute Care Surg. 2013;74(6):1528-33.

21. Badawi O, Xinggang L, Hassan E, Amelung PJ, Swami S. Evaluation of ICU risk models adapted for use as continuous markers of severity of illness throughout the ICU stay. SCCM - 47th Critical Care Congress 25 - 28 Feb 20182018.

22. Deller D, Kunitz O. Wann kann der Patient von der Intensivstation verlegt werden? Praxis der Intensivmedizin. Berlin Heidelberg: W. Wilhelm, Spinger-Verlag; 2013. p. 342-7.

23. S-3 Leitlinie Analgesie, Sedierung und Delirmanagement in der Intensivmedizin (DAS-Leitlinie 2015) - Addendum -, (2015).

24. Juma S, Taabazuing M-M, Montero-Odasso M. Clinical Frailty Scale in an Acute Medicine Unit: a Simple Tool That Predicts Length of Stay. Canadian Geriatrics Journal. 2106;19(2):34-9.

25. Fernandez R, Serrano JM, Umaran I, Abizanda R, Carrillo A, J. L-PM, et al. Ward mortality after ICU discharge: a multicenter validation of the Sabadell score. Intensive Care Med. 2010;36(7):1196-201.

26. Bakker J, Damen J, van Zanten AR, Hubben JH, Protocollencommissie Nederlandse Vereiniging voor Intensive C. [Admission and discharge criteria for intensive care departments]. Ned Tijdschr Geneeskd. 2003;147(3):110-5.

27. Padilha KG, Stafseth S, Solms D, Hoogendoom M, Monge FJ, Gomaa OH, et al. Nursing Activities Score: an updated guideline for its application in the Intensive Care Unit. Rev Esc Enferm USP. 2015;49 Spec No:131-7.

28. Gajic O, Malinchoc M, Comfere TB, Harris MR, Achouiti A, Yilmaz M, et al. The Stability and Workload Index for Transfer score predicts unplanned intensive care unit patient readmission: initial development and validation. Crit Care Med. 2008;36(3):676-82.

29. NGUYEN Y-N, Peters J, Deng Y. Evaluation of Stability and Workload Index for Transfer (SWIFT) Score as a mechanism for determining critical care unplanned readmission during hospitalization in surgical patients. Future.1:4.

30. Sanson G, Marino C, Valenti A, Lucangelo U, Berlot G. Is my patient ready for a safe transfer to a lower-intensity care setting? Nursing complexity as an independent predictor of adverse events risk after ICU discharge. Heart & Lung. 2020;00:1-8.

31. Goldfrad C, Rowan K. Consequences of discharges from intensive care at night. The Lancet. 2000;355(9210):1138-42.

32. Gantner D, Farley KJ, Bailey M, Huckson S, Hicks P, Pilcher DV. Mortality related to after-hours discharge from intensive care in Australia and New Zealand, 2005–2012. Intensive Care Med. 2014;40(10):1528 - 35.

33. Guidance on the Provision of Intensive Care Services: UK Faculty of lntensive Care Medicine, London; 2015 [Available from: <https://www.ficm.ac.uk/sites/default/files/GPICS%20-%20Ed.1%20%282015%29_0.pdf>.
