# Supplementary material for: Delphi study to derive expert consensus on a set of criteria to evaluate discharge readiness for adult ICU patients to be discharged to a general ward—European perspective
Source: BMC Health Serv Res. 2022 Jun 13;22:773. doi: 10.1186/s12913-022-08160-6 (PMC9190161; doi:10.1186/s12913-022-08160-6)
Supplement: Supplementary file 2 — Additional file 2. [file 12913_2022_8160_MOESM2_ESM.docx]

**Delphi study to derive expert consensus on a set of criteria to evaluate discharge readiness**

**for adult ICU patients to be discharged to a general ward**

**- European perspective**

**Detailed online Delphi process description**

Maike Hiller MA^1,4^, Maria Wittmann, MD^2^, Hendrik Bracht MD PhD^3^, Jan Bakker MD PhD FCCM FCCP ^1,5,6^

1 Erasmus MC University Medical Center, Dept. of Intensive Care Adults, Rotterdam, The Netherlands

2 University Hospital Bonn, Dept. of Anesthesiology and Intensive Care Medicine, Bonn, Germany

3 Central Emergency Medicine Services and Department of Anesthesiology and Intensive Care Medicine, University Hospital Ulm, Ulm, Germany

4 Philips Medizin Systeme Böblingen GmbH, Dept. of Monitoring and Analytics, Clinical Services, Böblingen, Germany

5 New York University School of Medicine and Columbia University College of Physicians & Surgeons, New York, USA

6 Pontificia Universidad Catolica de Chile, Dept of Intensive Care. Santiago, Chile

Corresponding author: Maike Hiller, m.hiller@erasmusmc.nl

**Delphi method in general**

The Delphi process is a research tool of growing interest and application in healthcare, specifically in areas where there is limited scientific evidence, lack of agreement, incomplete knowledge or uncertainty (1) and conclusions are heavily relying on expert opinion. It was developed to facilitate formal discussion among selected experts and stakeholders around a particular topic, where they can exchange their knowledge or opinion anonymously about a complex topic. With a focus on a systematic collection and aggregation of informed judgment, the method encourages the participants to share and change their opinions – if needed - over several rounds of voting. The aim is to obtain a reliable group opinion and build consensus. The Delphi technique has four main characteristics that suited the objective of this study: anonymity between participants through a non-face-to-face format, iteration with controlled feedback of group opinion, statistical aggregation of group responses and expert input (2). Further, the method was selected for this study to build on the limited scientific evidence for established and well-defined ICU discharge criteria that was identified by the previously performed scoping literature review (3).

**1st round - Open Delphi round**

In the initial round, the panelists were asked to provide some demographic information before answering the first round of voting. After that, each expert reviewed each of the 40 criteria on the proposed list of discharge criteria, that was introduced with the provided pre-read document (online supplements a, doc. 1). Per criterion the possible actions for each panelist were:

- Don’t do anything, then the panelist agreed that the criterion will go to the next round.
- Comment on needed changes, then the investigators reviewed the change request entered by the expert and adapted the criterion or phrasing if needed for the next round.
- Comment with “remove” in case the panelist felt the criterion is not at all relevant. Then the investigators removed the criterion for the next round if ≥ 25% of the experts voted for removal.
- Add a criterion to the list, if the panelist thought, there was a relevant criterion missing in the proposed list. This criterion was then included in the next round.

After closing of the first round, the investigators analyzed, regrouped and restructured the results in order to derive an edited list for the second round.

**2nd round – Closed Delphi round**

The expert panel received the results of the open Delphi round. Each expert ranked each criterion using a 5-point Likert scale (“very relevant”, “relevant”, “cannot judge”, “not relevant”, “completely irrelevant”). At this stage, agreement for further inclusion of a criterion in the list was defined as answering either “very relevant” or “relevant” to the question. Criteria reaching ≥ 90% agreement were then automatically included in the final set. Criteria reaching < 75% agreement were excluded from further rounds. Criteria reaching 75% - 89% agreement went to the third round. For rejected criteria it was also reviewed if there might have been consensus in one of the panelists subgroups (clinicians/ nurses) and reflected among the investigators if the level of disagreement in the other subgroup would still justify exclusion.

**3rd round – Closed Delphi round**

The Delphi group received those criteria from the second round that reached 75% - 89% agreement. Earlier aggregated voting results were displayed behind each criterion. Participants were asked to review their vote in context of the votes of the complete panel and consider the displayed comments per criterion collected from the previous round. With that perspective, panelists could change their vote for certain criteria and add comments. The aim of this round was to provide outliers the option to change their opinion towards the group’s opinion based on collected arguments. However, changing votes or commenting was completely optional. The goal at the end of round 3 was to conclude on a list of criteria that all meet a consensus level of ≥ 90%. The consented criteria from the 3rd round plus the consented criteria with ≥ 90% agreement from the 2nd round formed the list of criteria that entered the 4th round of voting with the focus of fine-tuning each criterion on certain aspects.

**4th round- Closed Delphi round**

The Delphi group received the complete list of consented criteria, all with a consensus level of ≥ 90%, and was asked to complete the following five tasks on the set of criteria:

1. Agree on the phrasing of each of the criteria on the final list, including the proposal for the binary decision metric values for “Fit for discharge” and “Needs further intensive care therapy / monitoring” that were initially shared in the pre-view document. Panelists could select either “agree” or “don’t agree”. When having selected the latter, a change proposal via the comment field was mandatory.

2. For 18 criteria, select an appropriate time frame per criterion to indicate stability, preventing readmission within 48 hrs after ICU discharge. Selection options were 0-4 hrs., 4-8 hrs., 8-12 hrs., 12-24 hrs. > 24 hrs. Comments were optional.

3. For 9 criteria, select, which value would inform them best to evaluate discharge readiness. Selection options were “best value within defined time frame”, “worst value within defined time frame”, “mean value over defined time frame + X% of standard deviation” (here defining X via the comment field was mandatory), trend within evaluation time frame, other (here a comment was mandatory).

4. Indicate the importance per criterion to be met for discharge in context with the other listed criteria by selecting one of three options (“If this individual criterion is not met, it already prohibits discharge”, “If the criterion is not met, it already prohibits discharge, except e.g. certain illness patterns / patient groups” (here a comment on possible exceptions was mandatory), “Good if it is met”).

5. Select per criterion, who of the stakeholder group can evaluate best if the patient meets the discharge criterion. Selection options were “ICU clinician”, “ICU nurse”, “clinician from the receiving unit”, “nurse from the receiving unit”, “patient/ surrogate”, “other” (here a comment was mandatory).

All results and received comments from the 4th round built the basis for the 5th and final round. Based on received comments, some of the criteria were rephrased and simplified, values and ranges were edited, and grouping and deletion proposals of some criteria were included for a final round of iteration.

**5th round – Closed and final Delphi round**

In the 5th round, panelists were asked to go through the remaining three questionnaire pages and provide their vote on every criterion, and if required a comment. On the first page, they could either “agree” or “not agree” (here a comment was mandatory) with the proposed final list of criteria and the related binary decision metric. Second, the consented value calculation method was presented, and panelists could either “agree” or “not agree” (for “not agree” a comment was mandatory) with the proposed phrasing. Third, participants were asked to decide whether a criterion is “mandatory to be met” or “not mandatory to be met”. In case they selected “mandatory to be met”, but there should be exceptions for certain illnesses / patient groups, a comment needed to be provided.

Based on all voting results and received comments from the 5^th^ round, the final list of ICU discharge criteria was built.

**References**

1. Powell C. The Delphi technique: myths and realities. Journal of Advanced Nursing. 2003;41(4):376-82.

2. Trevelyan EG, Robinson N. Delphi methodology in health research: how to do it? European journal of Integrative Medicine. 2015;7:423-8.

3. Hiller M, Spohn K, Schütte JK, Bracht H, Hering R, Bakker J, et al. Objective patient transfer criteria and proactive transfer management to control ICU capacities. Anästhesie und Intensivmedizin. 2020;61(1):569 - 78.
