# Supplementary material for: Delphi study to derive expert consensus on a set of criteria to evaluate discharge readiness for adult ICU patients to be discharged to a general ward—European perspective
Source: BMC Health Serv Res. 2022 Jun 13;22:773. doi: 10.1186/s12913-022-08160-6 (PMC9190161; doi:10.1186/s12913-022-08160-6)
Supplement: Supplementary file 3 — Additional file 3. [file 12913_2022_8160_MOESM3_ESM.docx]

**Delphi study to derive expert consensus on a set of criteria to evaluate discharge readiness**

**for adult ICU patients to be discharged to a general ward**

**- European perspective**

**Detailed results description**

Maike Hiller MA^1,4^, Maria Wittmann, MD^2^, Hendrik Bracht MD PhD^3^, Jan Bakker MD PhD FCCM FCCP ^1,5,6^

1 Erasmus MC University Medical Center, Dept. of Intensive Care Adults, Rotterdam, The Netherlands

2 University Hospital Bonn, Dept. of Anesthesiology and Intensive Care Medicine, Bonn, Germany

3 Central Emergency Medicine Services and Department of Anesthesiology and Intensive Care Medicine, University Hospital Ulm, Ulm, Germany

4 Philips Medizin Systeme Böblingen GmbH, Dept. of Monitoring and Analytics, Clinical Services, Böblingen, Germany

5 New York University School of Medicine and Columbia University College of Physicians & Surgeons, New York, USA

6 Pontificia Universidad Catolica de Chile, Dept of Intensive Care. Santiago, Chile

Corresponding author: Maike Hiller, m.hiller@erasmusmc.nl

**Results**

**Fig. S1: Geographical distribution of participants in Europe, plus 2 participants (1 clinician, 1 nurse) from Canada**


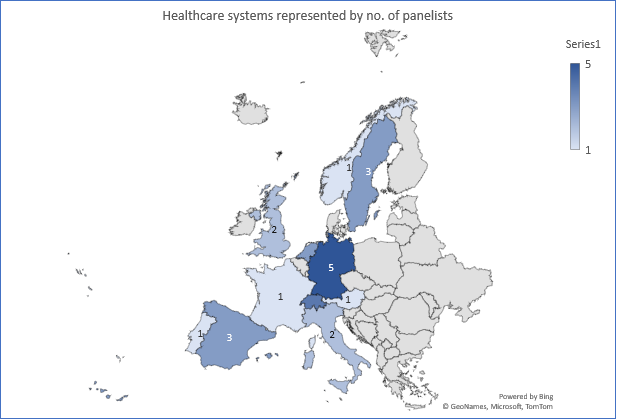


**Fig. S2: No. of panelists working in different types of ICUs Fig. S3: No. of panelists working per size of ICU**

**Fig. S4: No. of panelists per years of work experience Fig. S5: Panelists’ professional background**

**in an ICU environment**

**Tab. S2: Survey completion per voting round**

| **Rounds** | **Survey**  **completed** | **Survey**  **partially completed** | **Survey**  **not started** | **Total no. of panelists** |
| --- | --- | --- | --- | --- |
| **Round 1** | 26 | 1 | 1 | 28 |
| **Round 2** | 24 | 2 | 2 | 28 |
| **Round 3** | 21 | 2 | 4 | 27 |
| **Round 4** | 23 | 1 | 3 | 27 |
| **Round 5** | 23 | 2 | 2 | 27 |

**Detailed results description of all 5 rounds of voting**

**1^st^ round - Open Delphi round**

The first round was started with 28 panelists on June 24^th^, 2020 and ended on July 9^th^ with 26 completed survey, 1 partially completed and 1 not started survey. Each expert reviewed each of the 40 criteria on the proposed list of discharge criteria, that was introduced with the provided pre-read document (online supplements a, doc. 1).

After closing of the first round, the investigators analyzed, regrouped and restructured the results in order to come up with an edited list for the second round. To the initial 40 criteria, 20 criteria were added by the panelist. No criteria reached the threshold to be removed from the list (detailed results of round 1 in online supplement c, doc. 4, tab. S3).

**2^nd^ round – Closed Delphi round**

The Delphi group received the results of the open Delphi round with then 60 included items. Round 2 was open from 22^nd^ July 2020 until 10^th^ Aug. 2020 and closed with 24 completed, 2 partially completed and 2 not started surveys. 20 criteria reached consensus of ≥ 90%, 17 criteria got rejected from further rounds and 23 criteria reached agreement between 75% - 89% and were subject of the third round. For rejected criteria it was also reviewed if there might have been consensus in one of the panelists subgroups (clinicians/ nurses), which was the case for one criterion (“diastolic arterial blood pressure” with consensus among nurses with 91% and among clinicians with 44%). After reflecting among the investigators on the percentage distribution, it was decided to keep the exclusion rule for this criterion as the consensus among the clinicians was with 44% less than half compared to the nurses (detailed split of the votes on the Likert scale in online supplement c, doc. 4, tab. S4).

**3^nd^ round – Closed Delphi round**

The expert panel, for the remaining rounds with 27 participants as one opted out, received 23 criteria from the second round that reached 75% - 89% agreement. The goal was to achieve an inclusion/exclusion decision by asking each panelist to reflect per criterion on his own vote in context of the complete panel’s voting results and provided comments. Round 3 was open from 4^th^ Sept. to 18^th^ Sept. 2020 and was closed with a completion rate of 21 completed, 2 partially completed and 4 not started surveys. Out of the provided 23 criteria, 13 reached a consensus level of ≥ 90%. 10 criteria with a consensus level < 90% were rejected from further rounds. The 13 criteria from the 3^rd^ round plus the 20 criteria with ≥ 90% agreement from the 2^nd^ round formed the list of 33 criteria that entered the 4^th^ round of voting with the focus of fine-tuning each criterion on certain aspects (detailed split of the votes on the Likert scale in online supplement c, doc. 4, tab. S5).

**4^th^ round – Closed Delphi round**

The Delphi group received the list of 33 criteria, all with a consensus level of ≥ 90%, and were asked to agree on the phrasing of each of the criteria on the final list, now including the proposal for the binary decision metric values for “Fit for discharge” and “Needs further intensive care therapy / monitoring”. Further, they should indicate the importance per criterion to be met for discharge in context with the other listed criteria and who of the stakeholder group can evaluate best if the patient meets the discharge criterion. For a subset of 18 criteria, they were asked to select an appropriate time frame per criterion to indicate stability and for 9 criteria to select which value would inform them best to evaluate discharge readiness. The 4^th^ round was started on 4^th^ Nov. and closed on 25^th^ Nov. 2020 with 23 completed, 1 partially completed and 3 not started surveys. Upon completion, voting results and comments were reviewed and consolidated.

Agreement on binary decision metric

20 criteria reached consensus on the phrasing of ≥ 90% with only a few change comments, that were supportive to further improve the concrete phrasing of the criteria. The phrasing of 13 criteria received agreement between 63 and 88%. Here the comments helped to rephrase the criteria, include further aspects as e.g. relating the criterion to the abilities of the next lower level of care, change the threshold values and even regrouping several criteria into one more comprehensive criterion. For the 5^th^ round, 4 criteria got rephrased, for 4 criteria the values were refined, 5 criteria got proposed to be deleted, out of that for 4 criteria a more comprehensive criterion was proposed, and 20 criteria were not changed for round 5 (Further details on voting results on binary decision metric in online supplement c, doc. 4, tab. S6. Details on differences (round 4 results vs. round 5 set-up) in phrasing, values, and deleting proposals within tab. S7).

Criterion evaluation time frame

The perception of an ideal evaluation time frame per criterion was very diverse. Agreement within one of the proposed time frame categories was rather low with 42% as a maximum agreement level in only one criterion (“Do urine output, electrolyte level, and renal function allow patient discharge?”; for 12 – 24 hrs.; 42% agreement). The criteria “Blood oxygenation: SpO2 measures have been stable over defined time frame?” (for 4-8 hrs.; 38% agreement) and “Lung function: PaO2/ FiO2(alternatively SpO2 / FiO2) measures have been stable over defined time frame?” (for >24 hrs.; 38% agreement) have reached second highest agreement within a particular criterion evaluation time frame. Agreement of 33% was reached 8 times (“Respiratory rate”, “Pain therapy sufficient and feasible at the receiving unit?”, 4-8 hrs.; “Heart rate”, “Cardiac rhythm stable over defined time frame?”, “Hemoglobin value stable over defined time frame?”, “Active bleeding or risk of bleeding?”, “Hyperkinetic / hypokinetic delirium”, 12 – 24 hrs.; “Is the airway patent?”, “Confusion Assessment Method in Intensive Care (CAM-ICU)”, > 24 hrs.). All other criteria reached lower level of agreement within one evaluation time frame category (Further details on voting split among proposed evaluation times in online supplement c, doc. 4, tab. S6). Based on the very heterogeneous vote distribution and received comments, the investigators decided to exclude the survey on “criterion evaluation time frames” from further iteration in the 5th round and rather report and conclude on the results from the 4th round.

Criterion calculation method

Most of the panelists prefer to evaluate discharge readiness based on the trend within the evaluation time frame (8 out of 9 criteria reached an agreement for trend between 50 – 63%). In one criterion (“Cardiac rhythm stable over defined time frame?”), 50% of the panelists agreed on the worst value that would inform best to evaluate discharge readiness. For the other criteria, the worst value within the defined time frame was always the second-best option after trend with agreement between 17 – 33%. Based on the comments received and the split of the votes on mainly the proposed values “worst value” and “trend”, the investigators decided to combine the two values for 4 out of 7 criteria in round 5. The phrasing proposal for round 5 was then “Worst value must be within acceptable range AND trend must be stable over defined time frame” (Further details on voting split among proposed value calculation methods in online supplement c, doc. 4, tab. S6).

Criterion importance rank

When the panelists were asked to indicate the importance per criterion to be met for discharge in context with the other listed criteria (voting options: “If this individual criterion is not met, it already prohibits discharge”; “If the criterion is not met, it already prohibits discharge, except e.g. certain illness patterns / patient groups”; “Good if it is met”), 7 criteria reached ≥ 70% agreement on a particular voting option. 8 criteria reached agreement ≥ 70% with accumulated percentage distribution of the voting options “If this individual criterion is not met, it already prohibits discharge” and “If the criterion is not met, it already prohibits discharge, except e.g. certain illness patterns / patient groups”. However, 18 criteria reached agreement < 70%, when accumulating these two voting options. These results led the investigators team to enter the fifth round of voting with a more simplified wording for the criteria importance ranking. Participants could then select between the options “mandatory to be met” and “not mandatory to be met”. In case they had chosen “mandatory to be met”, and there should be exceptions for certain illness patterns / patient groups, those should have been provided via the comment field (Further details on voting split among the criteria importance ranking in online supplement c, doc. 4, tab. S6).

Decision maker per criterion

On the question, who of the stakeholder group can evaluate best if the patient meets the discharge decision criterion, ≥ 70% agreement was reached for 9 criteria:

- “Is the cough good and effective?”, 70% agreement on the ICU nurse
- “Lung function: PaO2 / FiO2 (alternatively SpO2 / FiO2) measures have been stable over defined time frame?”; “Cardiac rhythm stable over defined time frame?”; “Hypervolemia/ hypovolemia”; “Hemoglobin value stable over defined time frame?”; “Active bleeding or risk of bleeding”; “Do urine output, electrolyte level, and renal function allow patient discharge?”; “Therapeutic susceptibility: Patient doesn’t benefit from ICU care anymore and negative effects may outweigh”; “Patient no longer meets ICU admission criteria and meets admission criteria for a lower level of care”; 70 – 83% agreement on ICU clinician

24 criteria met agreement levels per decision maker < 70%. A general insight from the panelists’ comments and the voting distribution was, that a lot of criteria need to be evaluated by the interdisciplinary team, that often should even involve a clinician or nurse from the receiving unit. In the performed survey, there was no selection option for the interdisciplinary team. That is why panelists commented via the comments field on the need to have an interdisciplinary team (13 related comments). Based on very heterogeneous vote distribution and received comments, the investigators decided to exclude the survey on the “decision maker per criterion” from further iteration in the 5th round and rather report and conclude on the results from the 4th round (Further details on voting split among the preferred decision makers in online supplement c, doc. 4, tab. S6).

All results and received comments from the 4^th^ round built the basis for the 5^th^ and final round. Based on received comments, some of the criteria were rephrased and simplified, values and ranges were edited, and grouping and deletion proposals of some criteria were included for a final round of iteration (online supplement c, doc. 4, tab. S7).

**5^th^ round – Closed and final Delphi round**

In the 5th round, panelists were asked to go through the remaining three survey pages (criteria phrasing with binary decision metric; consented value calculation methods; criteria importance rank), provide their vote on every criterion, and if required a comment. Round 5 was open from 5^th^ to 21^st^ March 2021 and closed with 23 completed, 2 partially completed and 2 not started surveys.

Agreement on binary decision metric

Out of 33 presented criteria, 23 criteria reached > 90% consensus for the final (re-)phrasing. Proposed deletion for 5 criteria was agreed with > 90% consensus per criterion. 5 criteria reached 75% - 89% consensus on the final (re-)phrasing (Further details on voting split among the preferred decision makers in online supplement c, doc. 4, tab. S8). The investigators reviewed the provided comments and agreed on adaption of the phrasing of 4 criteria based on the comments (Phrasing comparison between round 5 proposal and final list, as well as the panelists’ change suggestions from round 5 (hidden columns) in online supplement c, doc. 4, tab. S9). The investigators decided that the final phrasing of the ICU discharge criteria should not go through another round of voting but rather be validated on appropriateness and practicality in an implementation study in daily clinical routine. For 3 criteria that received comments on further need for specification in round 5 (“Hypervolemia /hypovolemia: Does the current volemia status require ICU monitoring?”; “Hemoglobin value stable over defined time frame?”; both scored final consensus of 88%; “Can the neurological status of the patient be adequately handled and monitored at the receiving unit?”, 100% consensus level), the investigators decided that at this theoretical level, these criteria cannot be specified any further and implementation and testing in daily clinical practice should help further adaptation if needed.

Value calculation method

Out of the 7 provided criteria, 1 criterion was consented to be removed from the binary decision metric and thus also not being relevant in this category anymore. 5 criteria reached > 90% consensus (“Respiratory rate”, “Heart rate”, “Mean arterial pressure” for “Worst value must be within acceptable range AND trend must be stable over defined time frame”; “Cardiac rhythm stable over defined time frame?” and “Hemoglobin value stable over defined time frame?” for “Trend must be stable over defined time frame”). The criterion on “Blood oxygenation” reached 83% agreement on “Worst value must be above threshold value AND trend must be stable over defined time frame”. In addition, it got also change proposals for the phrasing and its threshold values which were realized for the final list (changed to “Blood oxygenation: SpO2 measures have been stable over defined time frame and patient is breathing on room air?” with changed “Fit for discharge” threshold value of “stable SpO2 ≥ 92% OR stable around lower patient individual baseline value”). One general comment was made several times on the value calculation method “Worst value must be within acceptable range…”: ‘There could easily be one or two values a little outside the "acceptable range" - depending on how "acceptable" range is defined. This is especially the case if there is continuous monitoring in place as measured values are not always accurate. But the patient could still be fit for discharge.’

Criterion importance ranking

Without the criteria proposed for deletion, 17 of the remaining 28 criteria reached > 90% consensus on “mandatory to be met”. 11 criteria reached 73% - 89% consensus on “mandatory to be met”. Looking at the voting alternatives, the highest vote for “not mandatory to be met” with 26% was reached for the criterion “Patient’s preference is to stop intensive care therapy and to leave the ICU”. Having reviewed the comments for this criterion, the investigators discussed that it wouldn’t be logic to enforce meeting this criterion. Rather should the care team reflect on the four medical ethical principles in this context (patient autonomy, beneficence, justice, and non-maleficence) and pay close attention whether discharge could be facilitated if the patient prefers to stop intensive care. Based on that, the criterion importance rank was changed to "not mandatory to be met" in the final list. Second highest scored the criterion “If discharge at night or weekend can’t be avoided, are measures in place to protect patient safety?” with 22% agreement for “not mandatory to be met”. Here, a panelist’s comment emphasized on that this criterion could be overruled in context of ICU capacity shortages and weighting the beneficence and justice aspects of a larger patient group (like during the COVID pandemic). However, as these should be exceptions, the criterion was left with the rank “mandatory to be met” in the final list.

Finally, over a course of 5 rounds of voting on a basis of 40 initial ICU discharge criteria, where several criteria have been added and deleted along the way through the panel’s consensus, the study resulted in 28 well defined ICU discharge criteria for adult patients, that should be applicable to any type of ICU.

**Doc. 4: Excel file with:**

Initial proposal

Tab. S3.) Results round 1

Tab. S4.) Results round 2

Tab. S5.) Results round 3

Tab. S6.) Results round 4

Tab. S7.) Phrasing comparison round 4 and 5

Tab. S8.) Results round 5

Tab. S9.) Phrasing comparison round 5 and final list

Tab. S10.) Final ICU discharge criteria list
